# Mucins and their receptors in chronic lung disease

**DOI:** 10.1002/cti2.1120

**Published:** 2020-03-17

**Authors:** Emma Denneny, Jagdeep Sahota, Richard Beatson, David Thornton, Joy Burchell, Joanna Porter

**Affiliations:** ^1^ Leukocyte Trafficking Laboratory Centre for Inflammation and Tissue Repair UCL Respiratory Rayne Institute University College London London UK; ^2^ Breast Cancer Biology Group Division of Cancer Studies King's College London Guy's Hospital London UK; ^3^ Wellcome Trust Centre for Cell‐Matrix Research School of Biological Sciences Faculty of Biology, Medicine and Health Manchester Academic Health Sciences Centre University of Manchester Manchester UK

**Keywords:** glycan‐binding protein, glycosylation, immunology, mucin, pulmonary

## Abstract

There is growing recognition that mucus and mucin biology have a considerable impact on respiratory health, and subsequent global morbidity and mortality. Mucins play a critical role in chronic lung disease, not only by providing a physical barrier and clearing pathogens, but also in immune homeostasis. The aim of this review is to familiarise the reader with the role of mucins in both lung health and disease, with particular focus on function in immunity, infection and inflammation. We will also discuss their receptors, termed glycan‐binding proteins, and how they provide an attractive prospect for therapeutic intervention.

## Graphical abstract

This review article explores the role of mucins in respiratory health and disease, with particular focus on their receptors, the glycan‐binding proteins.

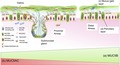

## Introduction

Mucus is one of the most ancient components of host defence. Its fundamental importance is emphasised by its conservation throughout the animal kingdom from snails to whales. The composition and function of mucus are much more complex than originally appreciated and are areas of growing interest and intrigue.

Mucus is a mixture of water, ions, glycoproteins, proteins and lipids.^1^ It plays a vital role in protecting the lungs from pathogens and toxins by forming the first line of innate defence in the respiratory tract. Mucins are major macromolecular components of mucus and are responsible for its chemical and physical properties.^2^ They are highly glycosylated proteins and are able to form a protective barrier as well as have a role in cell signalling, via interaction with their specific receptors, the glycan‐binding proteins (GBPs), on immune cells.

Although mucus and mucins are essential for airway defence, a number of diseases are associated with either abnormal mucus production, or alterations in mucin structure and glycosylation, which change the physical properties of the mucus barrier and the recognition of mucins by GBPs, thereby modulating the immune response. Mucin–GBP interactions protect the host by dampening and preventing excessive immune activation at the mucosal surface. Such homeostatic brakes have been co‐opted by pathogens and cancer cells for immune evasion and are increasingly recognised to play a key role in pathology.^1^

Further understanding of how mucins regulate the immune response in disease is a rapidly developing arena with the potential to identify novel therapeutic approaches.

## Mucins

Mucins are predominantly produced by epithelial cells, on the luminal surface, and their type and quantity vary depending on location. To date, 21 mucins have been identified, of which at least eight have been found to be expressed in the respiratory tract (Table 1). Mucins are classified into two groups: secreted and membrane bound. In the lung, the most prevalent secreted mucins are MUC5AC and MUC5B making up the majority (90%) of the mucin content of sputum, with the membrane‐bound mucins (MUC1, MUC4 and MUC16) making up the remaining 10%.^2^

**Table 1:** Mucins related to respiratory disease

Mucin	Chromosome	No. of amino acid repeats	Location of expression in respiratory tract	Respiratory disease association
Secreted – gel‐forming				
MUC2	11p15.5	16	Predominantly intestinal but a small amount is found in human lung	Asthma
MUC5AC	11p15.5	8	Upper respiratory tract predominant: trachea and bronchus	COPD, muco‐obstructive lung disease
MUC5B	11p15.5	29	Lower respiratory tract predominant: trachea and bronchus	IPF, RA‐ILD,
MUC19	12q12	7	Trachea	Unknown
Secreted – non‐gel‐forming				
MUC7	4q13.3	23	Saliva	Sjogren's, asthma
MUC8	12q24.3	41	Maxillary sinus mucosa	Chronic rhinosinusitis, asthma and COPD
Membrane bound				
MUC1	1q21	20	Upper and lower respiratory tract	Lung cancer, IPF, COPD
MUC4	3q29	16	Upper and lower respiratory tract	Lung cancer, COPD, muco‐obstructive lung disease
MUC16	19p13.2	156	Upper and lower respiratory tract	Lung cancer

### Mucin structure

Mucins are large glycoproteins which share common structural features consisting of a proline‐rich linear protein core and multiple O‐glycosylated carbohydrate side chains (glycans), producing a characteristic bottlebrush structure.^3^ The protein core comprises a variable number of tandem repeat (VNTR) domains rich in serine, threonine and proline (PTS) and is the primary site for O‐linked mucin‐type glycosylation.^4^ Glycans are covalently attached to the peptide backbone, predominantly by oxygen (O‐linked) via threonine and serine, and less commonly by nitrogen (N‐linked) via asparagine.^5^ N‐linked glycans are predominantly found outside the VNTR. In secreted polymeric mucins, the terminal regions are cysteine‐rich with little glycosylation and several conserved domains.^6^

### Glycosylation, glycans and glycan‐binding proteins (GBPs)

**Figure 1:**
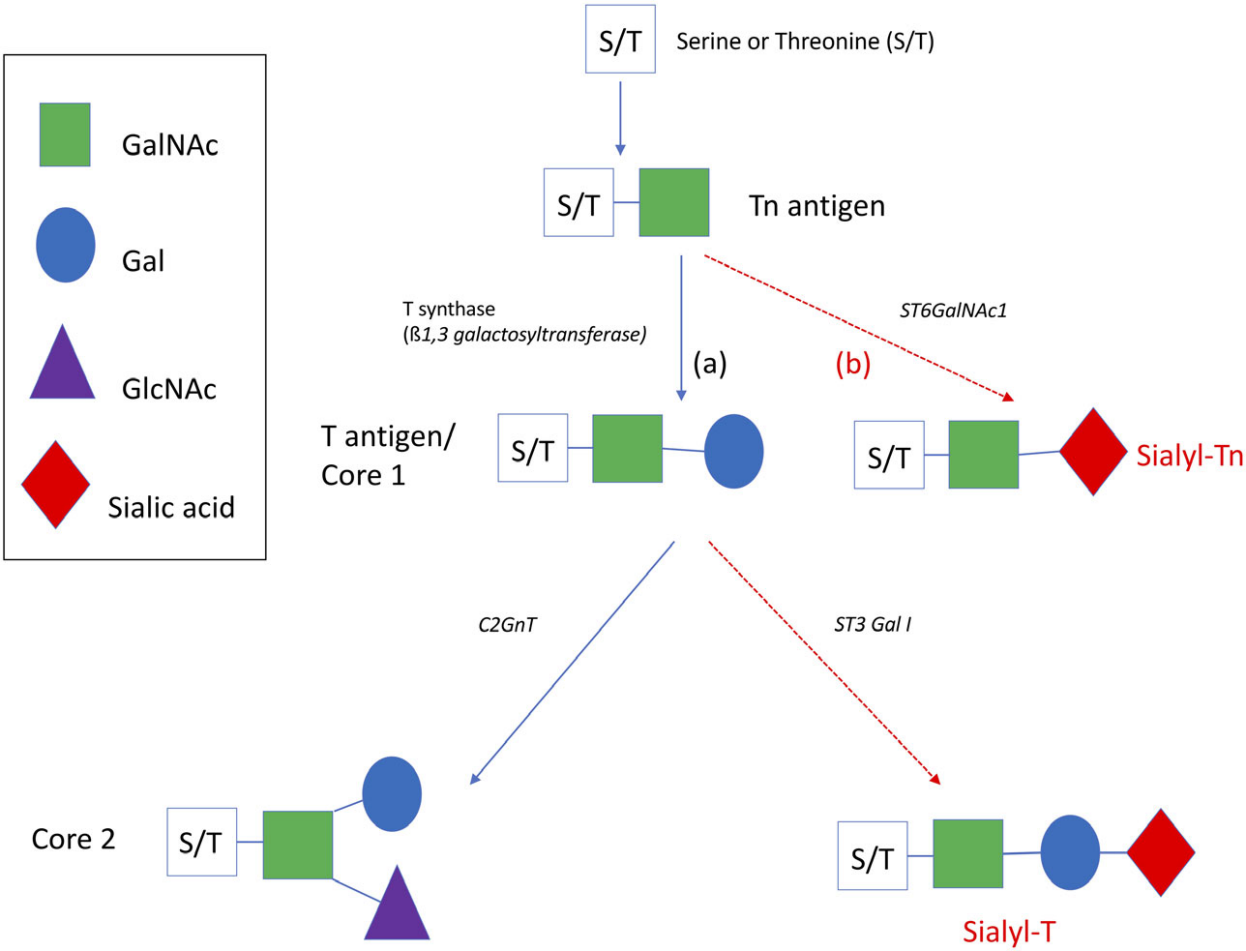
Glycan homeostasis in health and disease. **(a)** Schematic representation of the early stages of O‐linked mucin‐type glycosylation. **(b)** Schematic representation of aberrant glycosylation resulting in novel glyco‐epitopes and changes in O‐linked mucin glycosylation seen in disease (as indicated by the red arrows).

Glycans are vital to a number of cellular processes including adhesion, motility, inflammation, immunity and infection.^7^ Mucin‐type O‐linked glycosylation is initiated in the Golgi with the addition of N‐acetylgalactosamine (GalNAc), by a family of GalNAc transferases, to threonines and serines to form the Tn antigen.^8^, ^9^ The O‐linked glycans are then built up sequentially by the addition of simple sugars [e.g. galactose (Gal) and N‐acetylglucosamine (GlcNAc)] in various ordered combinations to form a number of core structures (Figure 1a). The addition of Gal to Tn by T synthase (core 1 β3‐galactosyltransferase) encoded by the *C1GALT1* gene forms the T antigen (core 1 structure). This enzyme requires the essential chaperone Cosmc for its correct folding in the ER.^8^ Further modification by adding GlcNAc to the T antigen forms the core 2 structure. The glycan side chains can be extended by accumulation of GlcNAc and Gal, depending on the tissue in which the cell resides. Such glycans can be further modified by processes such as fucosylation, sialylation and sulphation, which play a major role in the structure and function of the mucin.^5^, ^7^ It is clear that given such combinatorial variety, the glycans decorating any given mucin will vary in length, sequence and composition, which is key to their orchestration of the immune response to infection and their protection of the underlying epithelium against mechanical and chemical stress.^10^

These O‐glycans are recognised by the lectin family of GBPs that includes selectins, sialic acid‐binding immunoglobulin‐like lectins (siglecs) and galectins which are expressed on a range of immune cells and modulate the immune response.^7^

It is increasingly recognised that the ordered construction of glycan side chains may be dramatically modified during chronic lung diseases. In particular, the addition of sialylated, fucosylated or sulphated O‐glycans tends to be chain‐terminating, resulting in shorter glycans and leading to changes in the mucin physical properties and biochemical functions.^1^ In the tumor microenvironment, this has been shown to result in *de novo* glycan ligands for lectins on immune cells, often resulting in the activation of immunomodulatory pathways in these cells.^11^, ^12^ This is particularly relevant in cancers during which the glycocalyx of epithelial cells may transform to a structure made up of much simpler shorter glycans such as Tn or T that undergo sialylation to sialyl‐Tn and sialyl‐T, respectively^9^ (Figure 1b). At the same time, there is loss of epithelial cell polarity following an overexpression of the MUC1 cytoplasmic tail domain and inhibition of the Crumbs complex.^13^ Subsequently, mucins with repeated truncated glycans, no longer confined to the epithelial luminal surface, are expressed basally and can interact with receptors in the internal environment.

### The mucociliary escalator

**Figure 2:**
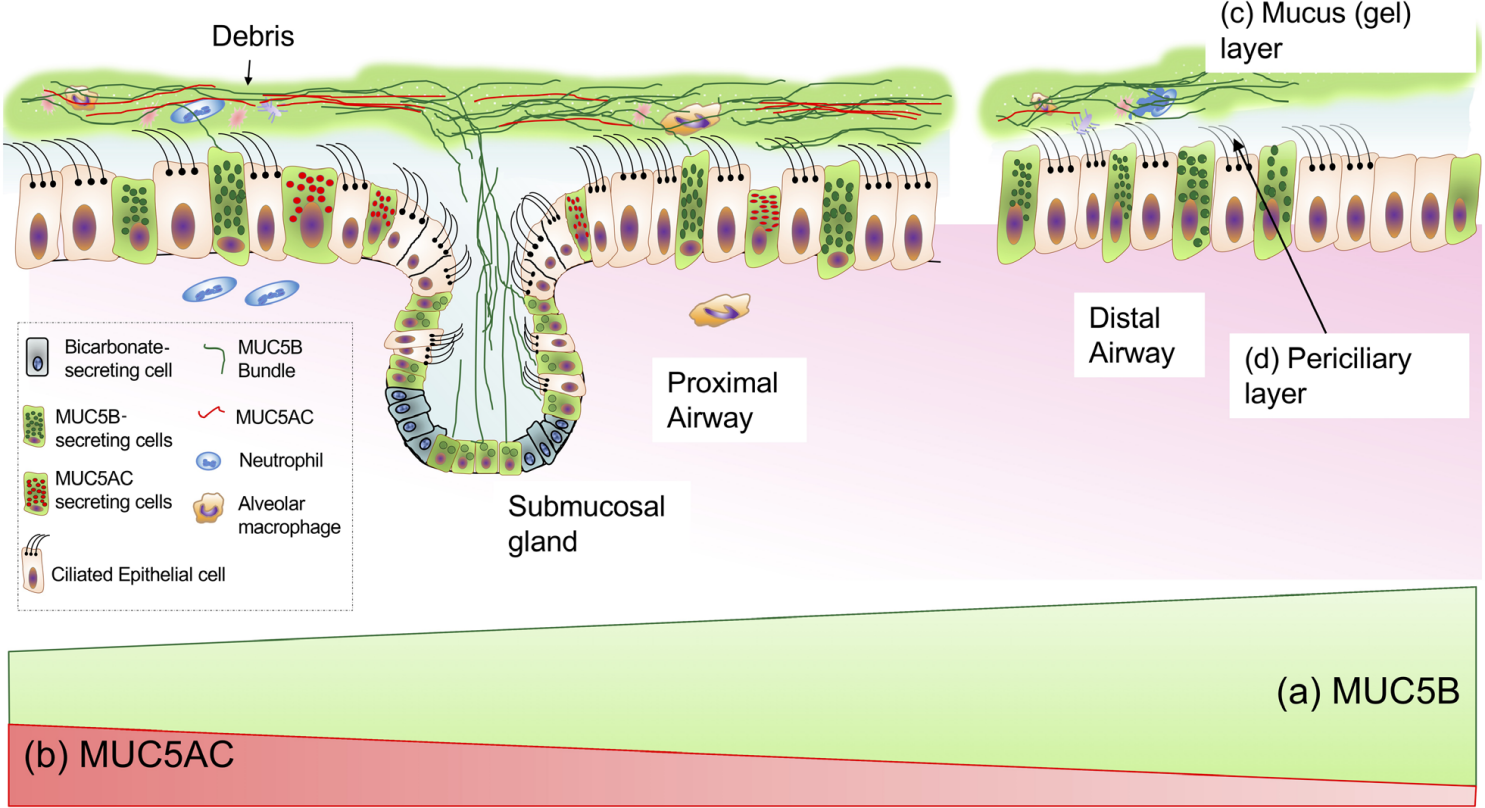
The mucociliary escalator. **(a)** MUC5B is the predominant mucin throughout the airway, and the majority is secreted distally. **(b)** MUC5AC is predominantly secreted in the proximal airway. **(c)** The mucus (gel) layer is made up of MUC5B, MUC5AC, water and ions. **(d)** The periciliary layer consists of the membrane‐bound mucins, water and ions.

The composition of normally secreted mucins is > 80% carbohydrate,^14^ allowing them to effectively sequester water molecules essential for the formation of mucus and the mucociliary escalator (Figure 2). Considering the average adult inhales 7–8 L min^−1^ of bacteria/fungal/debris‐laden air, the lungs remain surprisingly free from infection. This is achieved by the unique structural properties of MUC5B and MUC5AC allowing effective mucociliary clearance (MCC). Understanding the mechanism underlying this is evolving, and the current model is dependent on the existence of a complex interplay between two different hydrogel polymer layers and different mucin subtypes.^15^ The two hydrogel layers are made up of secretory and membrane‐bound mucins, respectively. The periciliary layer (PCL) consists of membrane‐bound mucins (MUC1, 4 and 16), and sitting above the PCL is the second hydrogel layer comprising the secreted mucins (MUC5B and MUC5AC).^16^ These two hydrogel layers can interact directly with each other, sharing water molecules and ions. In health, the membrane‐bound mucin PCL gel exerts a higher osmotic pressure than the MUC5AC/MUC5B hydrogel that overlays it, ensuring the environment surrounding the cilia is adequately hydrated and facilitating efficient cilia beating. This allows for debris trapped by the secreted mucin layer to be transported out of the airway by the motion of the cilia with minimal resistance.^16^

## Secreted airway mucins

In the normal lung, the secreted gel‐forming mucins, MUC5AC and MUC5B, are integral to the formation and function of mucus that lines airway epithelium, but their site‐specific expression is yet to be fully determined. Recent studies utilising RNA *in situ* hybridisation and immunohistochemistry in normal lung show that although MUC5B is constitutively secreted throughout the conducting airway except for the terminal bronchiole, the majority is produced in the distal airway by secretory epithelial cells.^17^ These cells together with submucosal glands are also responsible for MUC5B production in the proximal airway but to a lesser extent. In contrast, MUC5AC appears to be expressed predominantly in the proximal airway and is induced by a variety of triggers such as infection and cigarette smoke, as well as in allergic (type 2) inflammation, for example asthma.^18^ Cells either exclusively produce MUC5AC or MUC5B but not both, and MUC5B remains the predominant mucin throughout the airway.^17^

**Figure 3:**
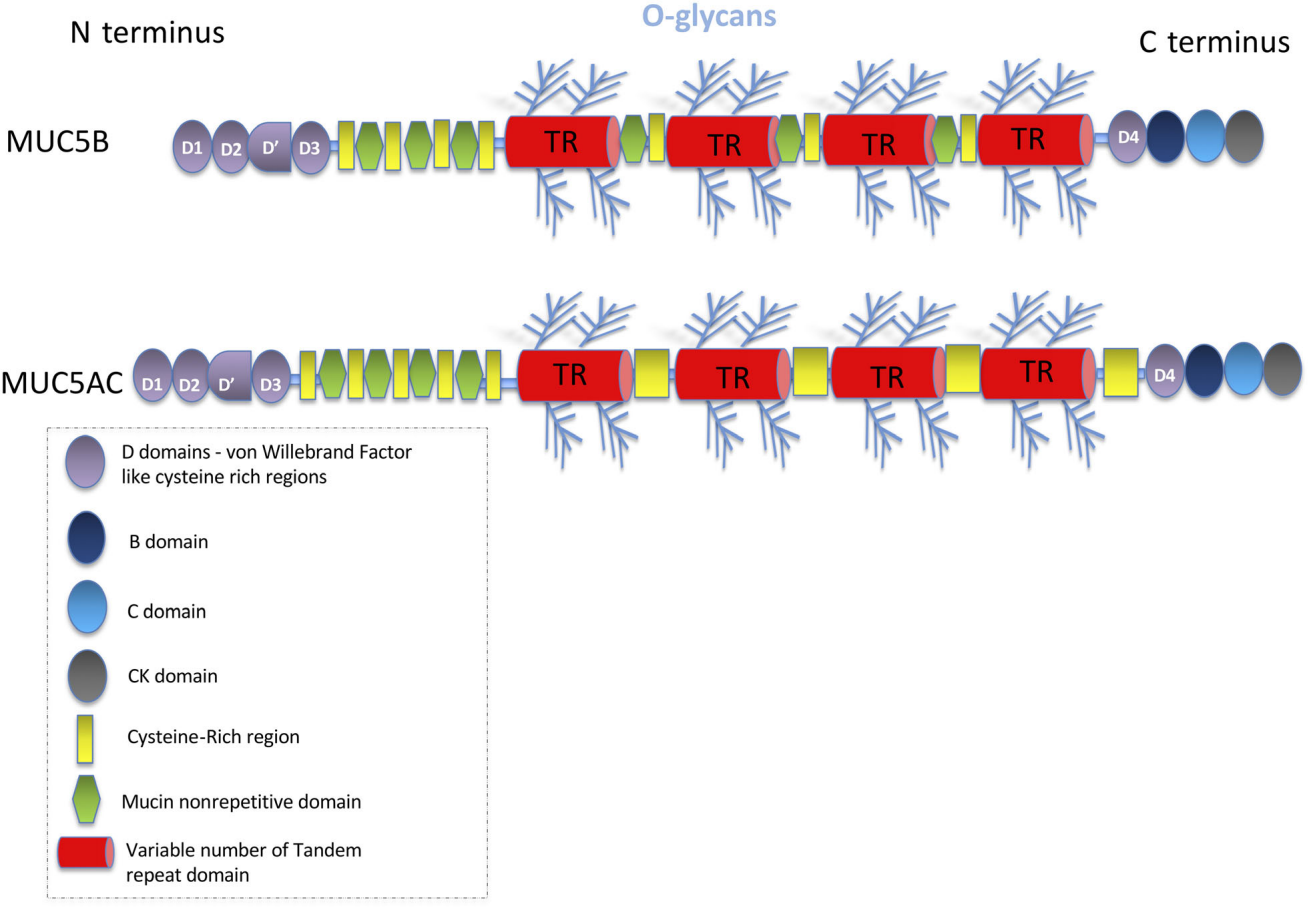
Structure of a secreted mucin. Schematic representation of a secreted mucin.

Secreted mucins are large glycoproteins that can have molecular weights in the region of 2–40 mDa. Cysteine‐rich von Willebrand factor‐like (vWF) D domains are found at either end, with D1, D2, D′, D3 found at the N terminus and D4 at the C‐terminal end^6^ (Figure 3). The mucins undergo post‐translational modification forming dimers in the ER, this occurs via disulphide bonds between the cysteine knot (CK domains) at their C‐termini.^19^ They are then transported to the Golgi where they are O‐glycosylated, further multimerised via disulphide bonding between D3 domains at their N termini and efficiently packed in a dehydrated and compact form into secretory vesicles. This process is dependent on noncovalent bonds formed by calcium ions between N‐terminal ends of dimers/polymers at acidic pH. When mucins are expelled from the secretory granule, the increased hydration and pH due to bicarbonate ions in the local vicinity result in the release of calcium ions holding the mucins in their compact conformation and their rapid volume expansion to form mucus.^19^

MUC5B is the predominant mucin in the mucus gel layer of the mucociliary escalator, and it forms a network to trap debris. The pore size is dependent on the level of concentration of this layer: the more concentrated it is, the smaller the pore size. Any pulmonary triggers that result in differing ratios of MUC5B and MUC5AC can result in excessively adherent mucus or reduced pore size of the mucus gel.^20^

In the trachea of pigs, long bundles of MUC5B have been seen, which can sweep along the airway and collect debris.^21^, ^22^ The submucosal glands located in the proximal airway may promote the formation of these long bundles. However, the majority of MUC5B is found in the distal airway which lacks submucosal glands, and the molecular organisation and mechanism of action of MUC5B in this location are under investigation.

This picture is further complicated by the observation that two glycoforms of MUC5B exist – a low‐charge form and a high‐charge form – which relate to the level of sialylation and sulphation of the attached O‐glycans. How these glycoforms affect the properties of the mucus gel is yet to be determined but the low‐charge form has been found in higher concentrations in respiratory diseases such as COPD.^2^, ^23^

The importance of MUC5B in MCC and immune homeostasis is further demonstrated by the *Muc5b* knockout mouse. These mice are prone to chronic bacterial infections, failure of inflammation resolution and reduced life expectancy.^24^

It is apparent that the secreted mucins display a range of properties geared towards host defence, providing not only a physical barrier, but are potentially able to broadly influence the immune response through specific interactions with GBPs, and in particular with lectins expressed on diverse immune cells. Future work aims to clarify the role that mucins may play in bridging the innate and adaptive arms of the immune system.

## Membrane‐bound mucins

The predominant membrane‐bound mucins in the lung are MUC1, MUC4 and MUC16. These are membrane‐anchored glycoproteins expressed at the surface of respiratory epithelia, which span the cell membrane and do not form gels.^25^ MUC16 is the largest followed by MUC4 and then MUC1.^25^ However, MUC1 is the most extensively studied in the airway to date and will be the main membrane‐bound mucin discussed in this section. As work is done on the other mucins, it is possible that similarities will be identified.

**Figure 4:**
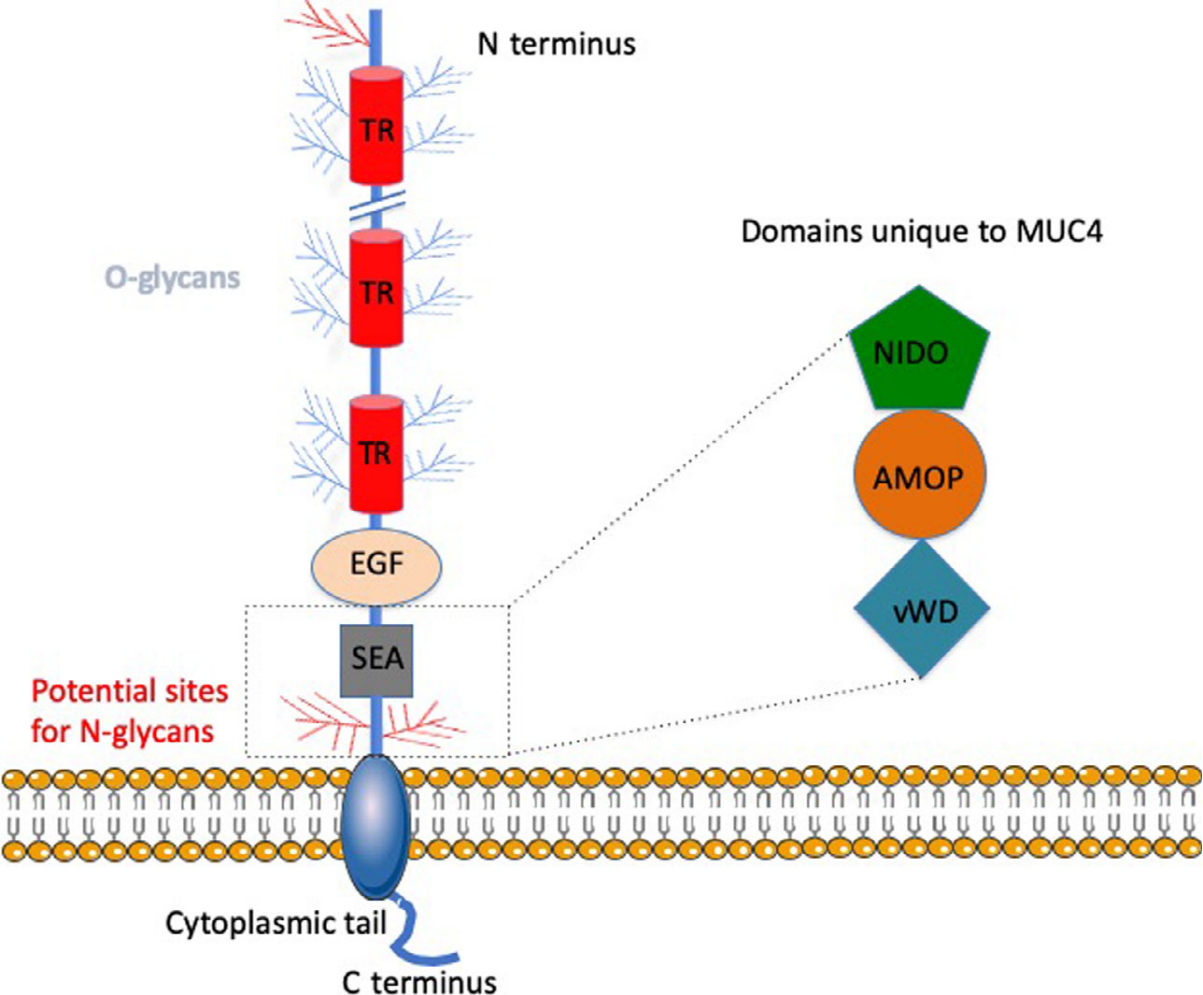
Structure of a membrane‐bound mucin. Schematic representation of a membrane‐bound mucin demonstrating the core structure and the domains unique to MUC4.

The membrane‐bound mucins consist of various domains (Figure 4): the extracellular O‐glycosylated VNTR which protrudes at the cell surface presenting a glycoarray into the extracellular space and participates in a wide range of cell interactions and cellular signalling pathways^26^; the transmembrane domain and a cytoplasmic tail (CT) domain. Both are involved in activation of intracellular signal transduction pathways, control of inflammation, and regulation of cell differentiation and proliferation.^27^ The amino acid sequence and phosphorylation sites in these domains lead to a range of functions in cell signalling.^28^

The extensive glycosylation of these mucins, not only serves as recognition sites for specific GBPs, but also regulates the biological properties of epithelial cells,^28^ whereby this hydrophilic environment is ideal for hydration and lubrication of the epithelia.^29^ The glycans extend further from the cell surface than most other extracellular receptors, which provides a barrier to the epithelium below, as well as a way for mucins to disrupt the adhesion of cells and pathogens.

MUC1 contains a single and MUC16 contains multiple sea urchin sperm protein enterokinase and agrin (SEA) domains. SEA domains are involved in protection against mechanical force and mucin degradation.^27^, ^28^ These are in the extracellular region of the mucin; they are able to self‐cleave and provide sites for glycosylation. The extracellular domain of MUC4 contains three epidermal growth factor (EGF)‐like domains that regulate signalling related to growth, motility and differentiation of the cell via interaction with EGF receptors.^28^ These domains contain conserved cysteine residues which may play a role in homodimerisation or oligomerisation of the mucin with itself as well as with other members of the mucin family.^30^ MUC4 also has the von Willebrand factor D‐like (vWF‐D) domain as well as two further unique domains, nidogen‐like (NIDO) and adhesion‐associated domain in MUC4 and other proteins (AMOP), which have been linked to cell adhesion, migration and angiogenesis.^28^ The various domains are listed in Table 2 (adapted from Corfield 2015^4^).

**Table 2:** Respiratory mucins and their domains (adapted from Corfield 2015^4^)

Domain	Domain function	Mucin	Mucin type
PTS – tandem repeat	Site for O‐linked glycosylation	All mucins	Secreted and membrane bound
Signal sequence at N terminus	Mediates secretion or membrane delivery	All mucins	Secreted and membrane bound
Cysteine rich and CYS domains	Enable mucin–mucin interactions	MUC5AC, MUC5B	Secreted
Cysteine knot	Dimerisation	MUC5AC, MUC5B	Secreted
Von Willebrand Factor D3	Mediates oligomerisation	MUC5AC, MUC5B	Secreted
Von Willebrand Factor D4	Contains GDPH autocatalytic cleavage site	MUC4, MUC5AC	Secreted and membrane bound
Cytoplasmic tail	Contains phosphorylation sites involved in signalling	MUC1, MUC4, MUC16	Membrane bound
SEA	Contains autocatalytic proteolytic cleavage site	MUC1, MUC16	Membrane bound
EGF	Mediate interactions between mucin subunits and ERBB receptors	MUC4	Membrane bound
Transmembrane	Membrane‐spanning sequence typical for membrane proteins	MUC1, MUC4, MUC16, MUC20	Membrane bound

Membrane‐bound mucins are cleaved post‐translationally into two subunits but remain associated throughout intracellular processing and insertion into the membrane by noncovalent bonds.^27^ They are also able to shed their extracellular domain via cleavage sites in their penultimate and/or last SEA domains and release soluble forms into the external environment. It is thought this process is triggered by phosphorylation events in the intracellular CT domain. The link between binding and shedding of the extracellular domain and activation of the intracellular domain is not proven; however, it has been postulated that the glycosylated extracellular domain senses the external environment and can feedback via the intracellular domain to stimulate essential mucosal maintenance and repair pathways.^27^

As well as being produced in the lung, MUC1 is also expressed in the mammary gland, female reproductive system, gastrointestinal tract and at lower levels by some immune cells. Levels increase dramatically during pregnancy and lactation, and a soluble form has been detected in breastmilk, peripheral blood, urine and the supernatant of cultures from MUC1‐positive cancer cell lines.^1^, ^29^ Shedding can be stimulated by proteases such as neutrophil elastase (NE). Shed MUC1 may form a gel, act as a decoy for pathogens, contribute to mucus obstruction, shield cancerous cells from the immune response or bind to and activate lectins on other cells.^31^

The CT domain of MUC1 contains several potential sites for kinase‐mediated phosphorylation and cell signalling. Six of the seven tyrosine residues are conserved across species suggesting functionality. The MUC1 CT domain can associate with various growth factor receptors including fibroblast growth factor receptor 3 (FGFR‐3),^32^ platelet‐derived growth factor receptor (PDGFR), epidermal growth factor receptor (EGFR) and other ErbB family members.^33^ Binding of ligands to these receptors increases their association with MUC1, stimulates phosphorylation of the MUC1 CT domain and results in intracellular signalling. The majority of such growth factor receptors would be expressed basally and would normally be separated from MUC1 by epithelial tight junctions, so that they would only be able to associate following damage to epithelial integrity or a loss of apical–basal polarity as is seen in cancer cells. MUC1 is not only able to influence cellular growth but also the local stroma and angiogenesis by increasing the levels of neuropilin‐1 (NRP1), a coreceptor of vascular endothelial growth factor (VEGF) and its ligand VEGF.^34^ It is clear that these events have potential roles, not only in the physiology of normal epithelial homeostasis and repair, but also in malignant cell growth and progression,^35^ and as such are being targeted in clinical trials.^36^

## Mucins and their interacting glycan‐binding proteins

Both membrane‐bound and secreted mucins are able to present glycan ligands for recognition by GBPs on immune cells. It is believed that GBPs recognise such mucin ligands via motifs that encompass both discrete O‐linked glycan structures and elements of their underlying scaffolds.

The GBPs are a large family that includes the lectins, selectins, siglecs and galectins, and we discuss each of these in turn in this review.

### Selectins

The selectins, consisting of E‐, L‐ and P‐selectin, are a family of single‐pass transmembrane cell adhesion proteins with an outer carbohydrate recognition domain that are widely expressed on endothelium, leucocytes and platelets. The selectins play a key role in leucocyte trafficking out of the circulation and into the tissues. However, the selectins can also bind O‐glycans expressed on mucins in luminal organs including the lung.^37^ The minimum structure for selectin recognition is a tetrasaccharide sialyl Lewis X (SLe^x^/CD15s) or its isomer Sialyl Lewis A (SLe^A^/CD19), containing sialic acid, fucose, Gal and GlcNAc. SLe^A^ is recognised as the circulating tumor marker CA19.9, which may be found in the O‐linked glycans on MUC1, MUC5AC and MUC16.^38^ Invasion of tumor cells coupled with a loss of epithelial cell polarity is thought to underlie basal release of the normally luminally expressed form into the circulation. This derangement of mucin expression of selectin ligands results in abnormal cell adhesion, implicated in the haematogenous spread of tumor metastasis and cancer‐associated thrombosis, increased angiogenesis and an altered immune recognition of and response to the mucin expressing cells.^29^ The reader is referred to an excellent review of this subject.^38^

Another scenario that results in selectin ligand expression on airway mucins is during inflammation. In one striking example, pyocyanin from *Pseudomonas aeuroginosa* (PA) upregulates SLe^x^ on mucins expressed by the bronchial epithelium to allow the bacterium to adhere and invade.^39^ However, although both cancer and inflammation are associated with abnormal mucin glycosylation, what is much less clear is whether there are physiological selectin ligands expressed on mucins in the healthy lung that play a role in normal lung homeostasis.

### Siglecs

The second GBP lectin family are the siglecs that are differentially expressed on restricted subsets of immune cells. There are 14 different mammalian siglecs but the identification of ligands for each individual family member is difficult because of their broad and overlapping affinities.^40^ Once again the physiological ligands for these GBPs are largely unknown but are thought to include sialoglycans on mucins. The siglecs are immunomodulatory receptors and ligand engagement usually results in reduced inflammation though immune inhibitory sequences (ITIMs) on cytoplasmic tails of most siglecs^7^ to mediate immune cell death, inhibit immune mediator release and/or enhance anti‐inflammatory mediator release. For example, antibodies or synthetic ligands are able to induce apoptosis of eosinophils, neutrophils and depletion of B cells through Siglec‐8, Siglec‐9 or Siglec‐2 (CD22), respectively. Although in general siglecs are inhibitory, there are certain siglecs, Siglec‐4 and Siglec‐14 to Siglec‐16, that do not have an ITIM or ITIM like motif, but instead signal through the association of DNAX activation protein (DAP)12.^41^ These are called activating siglecs as they promote immune activation through p38 MAPK and AKT signalling pathways. In some cases, activating and inhibitory siglecs are found on the same cell and thereby balance the immune response.

Probably the most important siglecs to be considered in chronic lung disease are Siglec‐8 on eosinophils, mast cells and basophils, and Siglec‐9 on neutrophils, monocytes and some T cells. Siglec‐8 and Siglec‐9 have been shown to play a role in the resolution of ongoing inflammation in asthma and COPD, respectively, with polymorphisms of *SIGLEC8* and *SIGLEC9* associated with disease severity.^40^, ^42^ Circulating levels of soluble Siglec‐9 have been proposed to impair disease resolution in COPD patients by ‘mopping‐up’ ligands for Siglec‐9 that would otherwise tone down inflammatory responses of alveolar neutrophils. Pathogens, specifically group B streptococcus (GBS) and PA, as well as tumor cells have developed mechanisms of sialoglycan overexpression to escape immune surveillance in an MHC class I‐independent fashion, through Siglec‐9 engagement.^43^, ^44^, ^45^ These observations have led to the suggestion that siglec‐directed therapies, particularly involving Siglec‐8 and Siglec‐9, may be appropriate for inflammatory, autoimmune, allergic and infectious diseases.^46^ However, much is still left to understand the role of endogenous airway sialoglycans in promoting siglec‐mediated immune regulation and the role of mucins in these events.

### Galectins

A third class of GBP lectins are the galectins that are expressed by many immune cells including activated macrophages, dendritic cells, and B and T cells. They bind galactose‐terminated oligosaccharides on mucins, to direct immune cell maturation, survival and activation.^7^ Galectins have also been implicated in tumor cell adhesion and progression, immunity, inflammation and wound healing. Galectin (Gal)‐1 demonstrates both pro‐ and anti‐inflammatory features that are not fully understood and has been shown to interact with MUC16.^28^ Gal‐1 agonists are under development as anti‐inflammatories but there are no clinical studies at the moment in chronic lung disease. The most well‐described galectin in relation to mucins is Gal‐3 that can interact with MUC1, MUC4 and MUC16 to alter cell surface polarisation, enhance tumor cell homotypic aggregation and increase growth factor signalling pathways.^28^, ^47^ The observation that the Gal‐3 knockout mouse is resistant to bleomycin‐induced lung fibrosis,^48^ and Gal‐3 is highly expressed in fibrotic tissue, suggested Gal‐3 as a potential therapeutic target in fibrotic lung disease. Although the precise mechanism by which Gal‐3 promotes fibrosis is ill‐defined, it has been shown to promote TGF‐β1‐mediated signalling in fibroblasts *in vitro*. Pulmonary fibrosis is associated with aberrant forms of MUC‐1; for example, the truncated MUC1‐ST (see below), which has fewer glycosylated branches and may expose ligand binding sites for Gal‐3, or via steric effects, makes interaction more accessible. Subsequently, this may result in increased transduction of profibrotic signalling pathways hitherto concealed. Two Gal‐3 inhibitors have been developed with this in mind: the modified disaccharide TD139a (Galecto Biotech) and a modified naturally occurring large galactose‐containing carbohydrate polymer GR‐MD‐02 (Galectin Therapeutics). Neither drug is absorbed orally and must be given intravenously (GR‐MD‐02) or inhaled (TD139), and both have a similar protective effect in a mouse model of lung fibrosis.^49^ A phase II study is currently being planned to evaluate the efficacy and safety of TD139 by dry powder inhalation over 52 weeks in patients with idiopathic pulmonary fibrosis (IPF).

In summary, the lectin group of GBPs recognise glycoproteins and their scaffolds to initiate and regulate ongoing inflammatory responses and are essential for homeostasis. However, tumor cells^9^, ^11^ and pathogens^50^, ^51^ are able to alter their glycans to engage lectins and so downregulate and evade the immune response.

## Disease associations

### Mucins in pulmonary fibrosis

Secreted and membrane‐bound mucins are both implicated in the development of fibrotic lung diseases. IPF is a rapidly progressive interstitial lung disease (ILD) which radiologically appears as honeycomb cysts (HC). A similar disease occurs in rheumatoid arthritis termed rheumatoid arthritis‐associated ILD (RA‐ILD).

The single greatest risk factor, genetic or otherwise, for the development of IPF and RA‐ILD is a gain of function, single nucleotide polymorphism (SNP) in the promoter region of the *MUC5B* gene (promoter variant rs35705950).^52^, ^53^ The wild‐type guanine (G) is switched for the thymidine (T) nucleotide (risk allele). This variant has a dose‐dependent effect, with those individuals heterozygous for the risk allele (GT) 4.5 times more likely to develop the disease, increasing to twenty times more likely if homozygous for the risk allele (TT). This SNP rs35705950 results in increased production of structurally normal MUC5B with an identical amino acid sequence to the wild‐type G version. It is associated with the development of HC. Recent data suggest that in IPF, MUC5B is not restricted to the conducting airways but co‐expressed with surfactant protein C in type 2 alveolar (AT2) cells and epithelial cells lining the HC.^54^, ^55^ Despite the propensity to develop IPF or RA‐ILD with this SNP, it appears to be somewhat protective with life expectancy extending beyond the usual 3–5 years, although a recent paper has challenged this association, suggesting that MUC5B actually decreases survival.^56^ The mechanism by which excess MUC5B results in IPF is unknown but current speculation involves impairment of the physical barrier resulting in adherent mucus that is difficult to expectorate and susceptible to colonisation by virulent bacterial strains. The finding that IPF patients have loss of heterogeneity of bacterial flora supports this theory.^57^ ER stress due to increased production and subsequent misfolding of MUC5B in distal epithelial cells may result in an impaired response to injury and development of fibrosis.

Krebs von den Lungen‐6 (KL‐6), an aberrantly glycosylated form of MUC1 which either carries the sialylated T antigen (MUC1‐ST) or a longer sialylated core 2 structure, or a mixture of both, shows increased levels of expression on airway epithelial cells in IPF.^58^ Injured AT2 cells release KL‐6, and measuring serum KL‐6 levels forms the basis of a biomarker used in Japan to assess disease progression and treatment response.^59^ The MUC1 intracellular cytoplasmic tail has also been shown to be activated in type 2 epithelial cells and fibroblasts both in animal models of pulmonary fibrosis and in the human disease. Phosphorylation of the cytoplasmic tail results in formation of a MUC1/beta‐catenin nuclear complex that promotes epithelial to mesenchymal and fibroblast to myofibroblast transition.^60^

We speculate that nintedanib, a novel antifibrotic and tyrosine kinase inhibitor, may achieve its therapeutic effect by reducing aberrant growth factor signalling by blocking the CT domain of MUC1 that may in part be enhanced by overexpression of MUC1‐ST in fibrotic lung.

### Mucins in asthma and COPD

Postmortem studies from patients with fatal asthma revealed death occurred from airflow obstruction because of extensive mucus plugging and bronchoconstriction. Mucus from patients with acute asthma has an increased proportion of MUC5AC and the low‐charge MUC5B glycoform. It is thought that MUC5AC together with smooth muscle contractility may be responsible for the airway hyperreactivity, as well as producing a hyperconcentrated mucus that is thicker, adherent and liable to form plaques and plugs.^61^

Muco‐obstructive plugs in COPD also consist of the low‐charge glycoform of MUC5B as well as MUC5AC. This hyperconcentrated mucus raft is more adherent, suppresses the cilia motion and arrests MCC. This results in repeated infections with mucus viscosity impairing the action of antimicrobial proteins secreted by the airway epithelial cell. These airways have elevated levels of NE, which correlates with disease severity and drives the development of bronchiectasis as NE‐induced mucus is more adherent.^62^

### Mucins in cystic fibrosis (CF) and non‐CF bronchiectasis

Normally, during short‐lived infections, increased mucus production is beneficial in dealing with the increased pathogen load, with cough acting as an important rescue mechanism to clear this excess mucus. The defects in CF mucus have led to a greater understanding of the protective role of mucus and MCC in airway infection. Thick mucus is the hallmark of CF and results from impaired mucus hydration.^14^ The CF transmembrane conductance regulator (CFTR) in goblet cells releases bicarbonate which increases pH and hence results in Ca^2+^ precipitating out and allowing mucins to be secreted into the lumen. In CF patients, defective bicarbonate release results in slower mucin unfolding and ultimately dehydrated and hyperconcentrated mucus. This occurs from birth and results in colonisation by oral anaerobes and eventually more virulent Gram‐negative species. It is this vicious cycle of persistent infection and inflammation that results in disease and loss of lung function. Non‐CF bronchiectasis likely results from a similar interplay between environmental stressors and inherent host defence defects resulting in bronchiectasis as the final common pathway.

### Mucins in infection and inflammation

The most common pathogens found in chronic respiratory diseases are *Pseudomonas aeuroginosa* (PA), *Staphylococcus aureus* and *Haemophillus influenza* and are associated with increased mucin production and mucus hypersecretion.^1^ These pathogens can activate MAPK pathways, which are important in transmitting extracellular signals from the cell surface to the nucleus to upregulate mucin genes.^1^

MUC1 is a receptor for PA where it has an anti‐inflammatory role; PA binds to the extracellular domain of MUC1 and triggers shedding of the extracellular domain which in turn induces phosphorylation of the CT domain to suppress Toll‐like receptor 5 (TLR) signalling.^63^ Furthermore, *Muc1* knockout mice show increased inflammation than the wild type, indicating an anti‐inflammatory role for MUC1 during airway infection.^63^

### Mucins in epithelial cancers

Overexpression and aberrant glycosylation of mucins have been a great focus of attention in epithelial cancers, such as lung, breast, ovarian and pancreas.^4^ The most frequently occurring cancer‐associated changes in glycosylation are increased sialylation which generates a terminal structure that cannot be extended, frequently involving Tn and T antigens and their sialylated counterparts – sialyl‐Tn and sialyl‐T antigens.^4^ These modified structures provide an enormous range of potential ligands for interaction with other receptors at the cell surface, which allows them to control the local microenvironment allowing tumors to grow, metastasise and invade, thereby evading the normal immune response.

Of the various mucins, MUC1 is predominantly found in lung adenocarcinoma and correlates with disease progression.^64^ The *Muc1* knockout mouse model has helped in understanding the critical role played by MUC1 in cancer pathobiology.^65^ During cancer development, epithelial cells lose their polarity resulting in reposition of MUC1 over the entire epithelial cell surface membrane, rather than just the apical surface. This means it can play a different role in cell–cell or cell–extracellular matrix interactions, not only by interacting with growth factor receptors expressed on the basal domain, but also with the intracellular adhesion molecule‐1 (ICAM‐1) via the protein component of its VNTR domains to facilitate tumor cell metastasis.^28^ MUC1‐ST can interact with siglecs expressed on leucocytes to affect downstream signalling pathways, which further amplify the permissive tumor microenvironment including T‐cell suppression and enhanced tumor cell growth.^11^ Interestingly, *Muc1* knockout mice display slower tumor progression and fewer metastases in multiple tumor models.^65^, ^66^

MUC4 can modulate cell apoptosis, regulate cell–cell adhesion and serve as a tumor marker or target for cancer therapy, most of which occurs through modulation of Erb family member signalling. Much work has been done looking at MUC4 overexpression in pancreatic cancer, where the NIDO domain is thought to play a key role in metastases.^67^ Through its extracellular EGF‐like domain, MUC4 interacts with the receptor tyrosine kinase, ErbB2, and controls ErbB2 and ErbB3 tyrosine phosphorylation. Overexpression of MUC4 is also seen in lung cancer, particularly in squamous cell carcinoma.^64^

The gel‐forming mucins are also involved in cancer. In lung adenocarcinomas, MUC5B and MUC5AC are strongly expressed, and MUC5AC specifically is associated with poorer survival where there is an associated *KRAS* mutation.^68^ In gastric cancers, MUC5AC expression is reduced, compared to the high expression of MUC5AC which usually characterises the healthy epithelial surface of the gastric tract, and this expression has been shown to be inversely associated with tumor stage including the extent of invasion and metastatic potential.^69^ MUC6 expression is also reduced in gastric cancers and is an independent predictor for malignant progression.^70^ MUC2 is the major structural component of colonic mucosa and has been associated with colorectal cancer, where its reduced expression results in increased inflammation and promotes IL‐6‐induced epithelial to mesenchymal transition.^71^

The CA125 antigen is a well‐recognised marker for ovarian cancer and corresponds to the cleaved extracellular TR region of MUC16. Mutations of MUC16 have also been associated with air pollution‐related lung cancer.^72^

Understanding the mechanisms leading to these biochemical and molecular changes of mucin structure in epithelial cell cancers may provide novel candidate biomarkers as well as potential therapeutic targets.

## Conclusion

In summary, mucins are complex glycoproteins with a multitude of both positive and negative effects in lung host defence. Although in mucus they form an essential physical barrier, their functions extend far beyond this with emerging roles in cell signalling, inflammation and cancer. The aberrant mucin glycosylation seen in tumor cells opens an expansive avenue of potential therapeutic targets including their receptors, the GBPs. Work has already begun on overcoming the immune checkpoint blockade by targeting siglecs and galectins in cancer. Beyond cancer, further understanding of mucin biology is critical in the search for novel treatments in a host of chronic lung diseases.

## Conflict of interest

The authors declare no conflict of interest.
